# Inverse correlation between E-cadherin and Snail expression in hepatocellular carcinoma cell lines *in vitro* and *in vivo*

**DOI:** 10.1038/sj.bjc.6600017

**Published:** 2002-01-07

**Authors:** W Jiao, K Miyazaki, Y Kitajima

**Affiliations:** Department of Surgery, Saga Medical School, 5-1-1 Nabeshima, Saga 849-8501, Japan

**Keywords:** hepatocellular carcinoma, cell line, Snail, E-cadherin

## Abstract

Hepatocellular carcinoma is a well-known malignancy in the world. However, the molecular mechanism of carcinogenesis and tumour progression remains unclear. Recently, reduced E-cadherin expression due to transcriptional suppressor Snail was proven in a panel of epithelial and dedifferentiated cells derived from carcinomas of various etiologies. In the present study, we examined Snail and E-cadherin mRNA/protein expression in five hepatocellular carcinoma cell lines with variable phenotypes (HuL-1, Hep-G_2_, Changliver, HLE, and HLF). The results demonstrated that the presence of Snail mRNA in HuL-1, Changliver, HLE and HLF cells detected by RT–PCR, which was further proven by *in situ* hybridization in tumours induced by HuL-1, Changliver, and HLF cells where Snail mRNA signals expressed in each of the sections. By contrast, E-cadherin mRNA and protein expression were only detected in Hep-G_2_ cells by RT–PCR and Western blot, respectively. These results were also consistent with the data obtained from *in vivo* immunohistochemical staining where membranous expression of endogenous E-cadherin protein was revealed only in tumour sections induced by Hep-G_2_ cells. Here we are the first to report that there is an inverse correlation between Snail and E-cadherin expression in HCC cells as well.

*British Journal of Cancer* (2002) **86**, 98–101. DOI: 10.1038/sj/bjc/6600017 www.bjcancer.com

© 2002 The Cancer Research Campaign

## 

Hepatocellular carcinoma (HCC), the major type of primary liver cancer, is one of the most common malignancies worldwide (Hussain and Harris, 2000; Yu *et al*, 2000). In Japan, HCC is one of the leading causes of cancer death (Okuda, 2000). Chronic hepatitis B virus and hepatitis C virus infections are the major contributing factors of hepatocarcinogenesis (Oda, 1999). However, the basic molecular mechanism of hepatocarcinogenesis remains unclear (Buendia, 2000; Durr and Caselmann, 2000).

E-cadherin (E-cad), one of the key cadherins, plays a major role in the establishment and maintenance of intercellular adhesion, cell polarity and tissue architecture (Takeichi, 1991). Abnormalities in expression and cellular distribution of E-cad are frequently associated with dedifferentiation, invasiveness, and lymph node or distance metastasis in a variety of human malignancies including primary HCC (Bracke *et al*, 1996; Endo *et al*, 2000; Takeichi, 1993). E-cad is also a critical factor in the process of intrahepatic metastasis of HCC (Osada *et al*, 1996). Thus, elucidating the mechanisms leading to disturb E-cad function in carcinomas has become a crucial issue because it is expected to provide new insights into the process of tumour invasion and consequently open avenues for therapy. It has been reported that E-cad expression is under strict spatiotemporal control and may be regulated at genetic (e.g. E-cad CDH1 gene mutation, LOH, (Kondoh *et al*, 2001) and/or epigenetic (e.g. CDH1 promoter methylation, SNP) (Kanai *et al*, 1997; Li *et al*, 2000) level. Most recently, there is an evidence that functional perturbation of E-cad expression during the process of epithelial dedifferentiation occurs at the transcriptional level (Hayashi *et al*, 1999; Hennig *et al*, 1996).

The transcriptional regulator Snail is the prototype of a family of zinc finger proteins that participate in various developmental and physiological processes (Cook, 1999). Snail expression in epithelial cells induces both epithelial-mesenchymal transitions and the acquisition of tumorigenic as well as invasive properties (Hemavathy *et al*, 2000). Most recently, Snail was described to contribute to repress transcription of E-cad gene by binding to the E-boxes of the CDH1 promoter (Giroldi *et al*, 1997). There is a strong inverse correlation between the Snail and E-cad expression in a panel of epithelial and dedifferentiated cells derived from carcinomas of various etiologies, including oral squamous carcinoma, breast, pancreas, colon, bladder cancer, melanomas, and fibroblast (Batlle *et al*, 2000; Cano *et al*, 2000; Yokoyama *et al*, 2001). However, the correlation of Snail and E-cad is not yet known in human HCC.

In the present study, we examined relationship between Snail and E-cad expression in five cell lines with variable phenotypes derived from human HCC. The results were unambiguously revealed that Snail inverses correlation with E-cad expression at mRNA and protein levels in HCC cells *in vitro* as well as *in vivo*.

## MATERIALS AND METHODS

### Cell culture and generation of tumours

Five established human HCC cell lines (HuL-1, Hep-G_2_, Changliver, HLE and HLF) (Chang, 1978; Dor *et al*, 1975; Huh and Utakoji, 1981; Knowles *et al*, 1980) were subjected to this study. All of these cells were obtained from RIKEN Cell Bank (Ibaraki, Japan). Cells were routinely cultured in Williams' medium E (W/E, ICN Biomedicals Inc, Costa Mesa, CA, USA) supplemented with 10% heat-inactivated FBS (JRH Biosciences, Lenexa, KS, USA), 100 μg ml^−1^ streptomycin, and 100 IU ml^−1^ penicillin, and incubated at 37°C under 5% CO_2_ in a humidified atmosphere. Tumours were induced in congenitally athymic male BALB/cA jcl nu/nu mice between 5 to 6-weeks-old by subcutaneous injection (5×10^6^ cells per injection site). Mice were purchased from CLEA (Tokyo, Japan), and housed in a laminar flow cabinet under specific-pathogen free conditions according to the institutional guidelines. Injected animals were observed every 3 days and sacrificed when the tumours reached an external diameter of 1.5–2.0 cm. The tumours were fixed in 10% formalin neutral buffered solution (WAKO, Osaka, Japan), paraffin-embedded, and 5 μm sections then cut and sequentially stained with H&E, and used for *in situ* hybridization (ISH) and immunohistochemical (IHC) staining.

### RT*–*PCR

Total RNA was isolated from each cell line using ISOGEN (Nippongene, Toyama, Japan). RT–PCR was carried out as described previously (Jiao *et al*, 2001) with specific primers. The primer pairs of human Snail (hSnail), E-cadherin (hE-cad) and GAPDH was designed as follows: hSnail (GenBank AF125377) forward 5′-TTC TTC TGC GCT ACT GCT GCG-3′ and reverse 5′-GGG CAG GTA TGG AGA GGA AGA-3′; hE-cad (GenBank Z13009) forward 5′-TCC CAT CAG CTG CCC AGA AA-3′ and reverse 5′-TGA CTC CTG TGT TCC TGT TA-3′; GAPDH (GenBank NM002046) forward 5′-TGG TAT CGT GGA AGG ACT CAT GAC-3′ and reverse 5′-ATG CCA GTG AGC TTC CCG TTC AGC-3′, respectively. The PCR product was an 883 bp fragment of hSnail gene, and a 502 bp fragment of the extracellular domain of the hE-cad gene. GAPDH was simultaneously amplified in each sample as the internal marker. All reactions were repeated at least twice.

### Western blot

Whole cell lysate without trypsin treatment was prepared from 5×10^6^ cultured cells using lysis buffer (0.1% SDS, 150 mM NaCl, 1 mM PMSF, 5 mM EDTA pH 8.0, 10 μg ml^−1^ trypsin inhibitor, 50 mM iodoacetamide, 50 mM Tris pH 7.4). After sonication and centrifugation, aliquots of each cell extracts containing equal amount of protein (50 μg) was resolved by 10% SDS–PAGE, and electrophoretically transferred onto Hybond™ ECL™ nitrocellulose membrane. Blots were probed with E-cad monoclonal antibody HECD-1 (1 μg ml^−1^, Takara Biochemicals, Tokyo, Japan), and developed with chemiluminescence as described previously (Jiao *et al*, 2001).

### Immunohistochemical staining

IHC staining on sections was performed essentially as described elsewhere (Jiao *et al*, 2001). HECD-1 was used at 1:250 dilutions. No staining was obtained when PBS was used instead of the primary antibody.

### Pretreatment of tumour sections for *in situ* hybridization

As a result of no satisfactory commercial specific antibodies against hSnail-peptide available yet, we developed an ISH method to detect endogenous Snail mRNA expression in tumour cells with an appropriate biotinylated hSnail cDNA probe. The 5 μm sections were mounted onto poly-L-lysine coated slides and dried at 37°C overnight. Sections were deparaffinized in xylene and dehydrated in ethanol. To obtain an optimal recovery of cells and proper removal of cellular protein for improvement of DNA probe penetration, a proteolytic digestion step was applied. The digestion was performed with proteinase K at a concentration of 10 μg ml^−1^ in 10 mM PBS (pH 7.4) for 20 min at 37°C. Sections were immersed in PBS containing 3% hydrogen peroxide to inhibit endogenous peroxidase activity.

### cDNA probe

To identify endogenous Snail gene expression in the sections, we designed a hybridizing specific cDNA probe, complementary to the sequence of the hSnail mRNA. The PCR primer for the hydbridization probe was: forward, 5′-AGG CTT GGG CCA AGT GCC CA-3′; reverse, 5′-GGT GGG CCC GCA GGT TGG AG-3′. The thermal cycle profile consisted of an initial denaturation at 94°C for 2 min, followed by 35 cycles with a 30 s denaturation at 94°C, a 30 s annealing of primers at 60°C, and a 1.5 min extension at 72°C, and a final 10 min extension at 72°C. The PCR product, corresponding to the complete hSnail cDNA sequence extending from 451–743 positions, was electrophoresed in 1.5% agarose gels. The 292 bp fragment in length of interest was then sliced cleanly under UV illumination, and recovered by using MagExtractor® DNA Fragment Purification Kit (TOYOBO Co., Ltd. Osaka, Japan). The cDNA probe was labelled with Biotin-16-dUTP using Biotin-High Prime Kit (Roche Molecular Biochemical, Mannheim, Germany) according to the manufacturer's instructions.

### *In situ* hybridization

ISH for Snail mRNA with appropriate positive and negative controls was performed. The probes were hybridized to the sections according to the standard protocol of the In Situ Hybridization Detection Kit (ISH-B1, Sigma). Twenty μl of Biotin-conjugated probe (1 ng 1 μl^−1^) were applied to the each slide under a coverslip. Denaturation was carried out by heating the slides in an oven to 95°C for 10 min. Hybridization was then performed for 16 h at 37°C in a sealed moist chamber. Coverslips were removed by submersing the slides in PBS. After blocking, the slides were applied of ExtrAvidin®-Peroxidase solution, the ISH signal was immunologically amplified using monoclonal anti-avidin-biotin conjugated solution followed by re-applying ExtrAvidin®-Peroxidase solution. Colour development was achieved by adding a freshly prepared substrate solution DAB. The sections were counterstained with haematoxylin, then dehydrated and mounted. We used poly d(T) Probe-Biotin labelled as a positive control and ISH solution as a negative control.

## RESULTS

In this study, four cell lines, i.e. HuL-1, Hep-G_2_, Changliver, and HLF, developed tumours in mice. However, HLF cells needed longer latency periods, grew slower, and reached an average size of less than 20% of the formers after 1 month. HLE cells had no tumorigenicity. Histological analysis of tumours derived from respective HuL-1, Changliver and HLF cells revealed dedifferentiated characteristics, whereas the tumour induced by Hep-G_2_ cells showed differentiated features (data not shown). All these histopathologic properties resemble those of the original tumour as reported elsewhere (Chang, 1978; Dor *et al*, 1975; Huh and Utakoji, 1981; Knowles *et al*, 1980).

**Figure 1:**
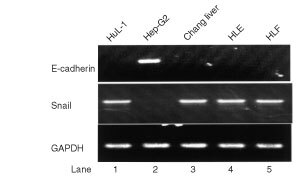
RT–PCR analyses of E-cad expression in Hep-G_2_ cells and Snail expression in HuL-1, Changliver, HLE, and HLF cells showed a strong inverse correlation between the expression of both mRNAs (upper and middle panels). A housekeeping gene, GAPDH, mRNA was simultaneously expressed in each sample (lower panels).

**Figure 2:**
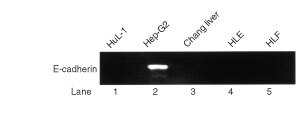
Detection of E-cad protein only in Hep-G_2_ cells was obtained on Western blot method with monoclonal antibody HECD-1.

To examine the expression of Snail and E-cad mRNA in these cell lines, RT–PCR analysis was performed. A clear inversed correlation between Snail and E-cad expression was observed (Figure 1

). Snail mRNA expression was found in HuL-1, Changliver, HLE and HLF cells but not in Hep-G_2_ cells, whereas E-cad mRNA expression was detected in Hep-G_2_ cells merely. In addition, we analyzed E-cad protein expression in these cell lines by Western blot method, E-cad protein was detected only in Hep-G_2_ cells as well with monoclonal antibody HECD-1 (Figure 2

).

**Figure 3:**
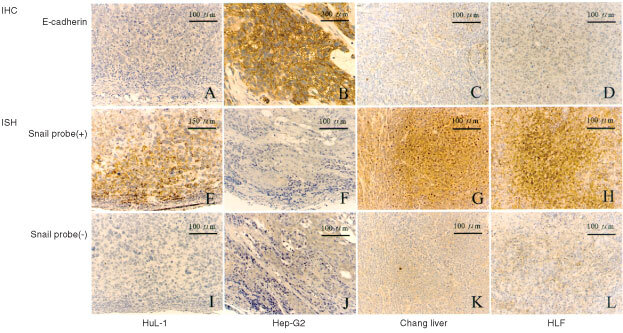
IHC staining of endogenous E-cad protein expression in the tumour sections induced by HuL-1 (**A**), Hep-G_2_ (**B**), Changliver (**C**) and HLF (**D**) cells (upper panels). Only Hep-G_2_ tumours clearly showed membranous expression of E-cad. ISH with biotinylated Snail probe detected positive brown staining in the cytoplasm of HuL-1 (**E**), Changliver (**G**) and HLF (**H**) tumour sections, representative of endogenous Snail mRNA expression, but not in Hep-G2 tumours (**F**) at all (middle panels). Lower panels (**I, J, K** and **L**) represented negative controls of each respective section for ISH.

By IHC staining of tumour sections, we found membranous expression of endogenous E-cad protein only in Hep-G_2_ cells (Figure 3B

). ISH with biotinylated hSnail probe detected positive brown staining in the cytoplasm of HuL-1, Changliver and HLF cells, but not in Hep-G_2_ cells at all. These *in vivo* IHC of E-cadherin expression and ISH of Snail mRNA expression staining patterns were consistent with the same expression profiles as found in their respective *in vitro* RT–PCR and Western blot analyses.

## DISCUSSION

HCC is known to have a poor prognosis because of multicentric development and intrahepatic metastasis (Ariizumi *et al*, 2001; Feitelson, 1998; Kojiro, 2000). However, the molecular mechanisms of hepatocarcinogenesis and intrahepatic metastasis are not yet well understood (Bergsland and Venook, 2000). Loss of both cell-cell adhesion and cellular differentiation is one of the characteristics of malignant cells, and this has been reported extensively to correlate with E-cad down-regulation (Bracke *et al*, 1996; Endo *et al*, 2000; Huang *et al*, 1999; Takeichi, 1993). Reduced E-cad expression due to transcriptional repressor Snail around CDH1 promoter region may participate in certain steps of carcinogenesis by reduction of intercellular adhesiveness, which may result in initiation of invasion and destruction of normal tissue morphology (Batlle *et al*, 2000; Cano *et al*, 2000). The present study demonstrated the presence of Snail mRNA in HuL-1, Changliver, HLE and HLF cells, but not in Hep-G_2_ cells detected by RT–PCR, these patterns were further proved by ISH in tumours, where Snail mRNA signals expressed in each tumour sections induced by HuL-1, Changliver, and HLF, respectively. By contrast, E-cad mRNA and protein expression were only detected in Hep-G_2_ cells by RT–PCR and Western blot analyses, these results were confirmed by the finding *in vivo* IHC staining where endogenous E-cad protein membranous expression was revealed only in tumour sections induced by Hep-G_2_ cells. To our knowledge, no reports have yet been published on the use of *in vivo* as well as *in vitro* molecular techniques to show that the zinc finger protein Snail has reversed correlation with E-cad at mRNA and protein levels in HCC cell lines. Our subsequent study will include expanding the number of HCC cell lines to further investigate the relationship between E-cad and Snail; Ectopic expression of Snail in hepatocytes to assess whether or not the transformed phenotype in HCC can be reversed by abrogating Snail function. Meanwhile, extending correlation analysis of Snail and E-cad expression patterns in HCC surgical specimens so as to evaluate whether Snail may be used as a screen for the invasion and progression of HCC in human is also in consideration.

Transcription factors critically regulate cell physiologic processes because they control the protein repertoire expression (Cook, 1999). Alterations in transcription factors have significant impact on disease and their treatment (Hedin *et al*, 2000). From the standpoint of hepatocellular carcinogenesis, unveil the mechanism of functions and regulation of Snail are not only important for understanding of hepatocarcinogenesis and intrahepatic metastasis processes, but will facilitate the design of novel therapies for HCC and the control of biologic responses after surgery (Guyton and Kensler, 1997; Hayashi *et al*, 1999).
